# Cued by What We See and Hear: Spatial Reference Frame Use in Language

**DOI:** 10.3389/fpsyg.2018.01287

**Published:** 2018-08-13

**Authors:** Kenny R. Coventry, Elena Andonova, Thora Tenbrink, Harmen B. Gudde, Paul E. Engelhardt

**Affiliations:** ^1^School of Psychology, University of East Anglia, Norwich, United Kingdom; ^2^Department of Cognitive Science and Psychology, New Bulgarian University, Sofia, Bulgaria; ^3^School of Linguistics and English Language, Bangor University, Bangor, United Kingdom

**Keywords:** reference frames, spatial language, visual cueing, eye movements, language production

## Abstract

To what extent is the choice of what to say driven by seemingly irrelevant cues in the visual world being described? Among such cues, how does prior description affect how we process spatial scenes? When people describe where objects are located their use of spatial language is often associated with a choice of reference frame. Two experiments employing between-participants designs (*N* = 490) examined the effects of visual cueing and previous description on reference frame choice as reflected in spatial prepositions (*in front of*, *to the left of*, etc.) to describe pictures of object pairs. Experiment 1 examined the effects of visual and linguistic cues on spatial description choice through movement of object(s) in spatial scenes, showing sizeable effects of visual cueing on reference frame choice. Experiment 2 monitored eye movements of participants following a linguistic example description, revealing two findings: eye movement “signatures” associated with distinct reference frames as expressed in language, and transfer of these eye movement patterns just prior to spatial description for different (later) picture descriptions. Both verbal description and visual cueing similarly influence language production choice through manipulation of visual attention, suggesting a unified theory of constraints affecting spatial language choice.

## Introduction

Talking about the world involves making choices regarding the words, phrases, and sentences to use. These choices are constrained by a number of different information sources. To start with, the world itself is described in only a finite number of ways, and these are a reflection of a limited number of different conceptualizations of it. For example, Garrod and Anderson (1987) found that interlocutors typically (implicitly) agreed upon particular ways of referring to object positions in arrays that conformed to four basic types of description schemas. Further, our choices are affected by what previous speakers have said about similar arrays and events. In dialog, it has been shown that speakers’ choices of syntactic and lexical representations (Branigan et al., 2000; Cleland and Pickering, 2003), gestures (Henderson and Ferreira, 2004), and choices for joint reference (Clark and Wilkes-Gibbs, 1986; Brown-Schmidt et al., 2005) are all affected by the previous behavior of an interlocutor. Moreover, linguistic choices – at least at the level of syntactic structure – are influenced by how attention is directed to a visual scene (Tomlin, 1997; Griffin and Bock, 2000; Gleitman et al., 2007). For example, when describing a picture of a man kicking a dog, participants were more likely to make a particular character the subject when that character was exogenously visually cued (i.e., man cued: *the man is kicking the dog*; dog cued: *the dog is being kicked by the man*) (Gleitman et al., 2007; Myachykov et al., 2012).

**FIGURE 1:**
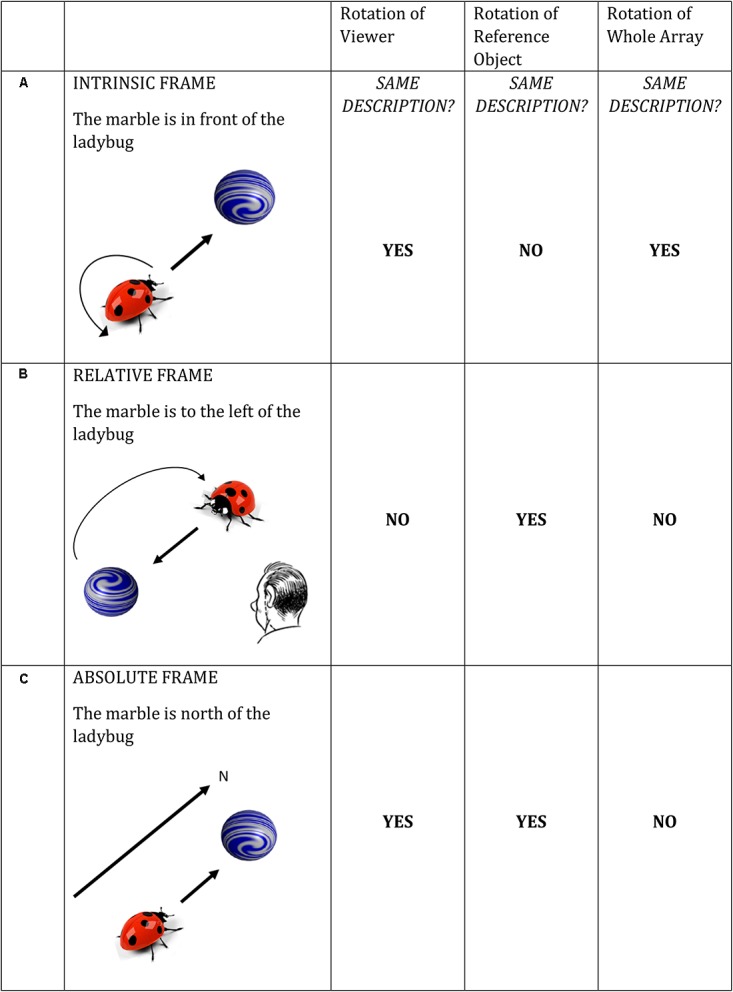
**(A–C)** Reference frames under conditions of rotation (adapted from Levinson, 1996).

Here we address the influence of (a novel form of) visual motion cueing and prior linguistic description on language production, with a view to further understanding *how* past constraints impact upon future language production. The test ground is communicating about the location of objects in space. Spatial communication requires selecting a spatial term from a range of available options, often associated with the choice of an underlying conceptual reference frame that guides the interpretation of spatial directions. Levinson (1996) differentiated three categories of reference frame: relative (viewer-centered), intrinsic (object-centered), and absolute (environment-centered). In **Figure 1A**, *the marble is in front of the ladybug* locates the marble using the intrinsic axis of the ladybug (i.e., intrinsic frame). In **Figure 1B**, *the marble is to the left of the ladybug* locates the marble with respect to the viewer looking at the picture (i.e., the relative frame). **Figure 1C** exemplifies an absolute reference frame based on a compass direction (*the marble is north of the ladybug*), which is infrequent in many languages and cultures (including English and German) in small-scale (table top) space. Levinson distinguishes these frames as a function of how different patterns of rotation affect changes in spatial description (within a given frame). For instance, rotating the ladybug by 180° (in **Figure 1A**) results in a different spatial preposition appropriate to describe the position of the marble, whereas changing the viewer position or rotating the whole scene (ladybug and marble) does not change the spatial description within the intrinsic frame. **Figure 1** spells out the effects of these three kinds of rotations for each reference frame.

Choice of spatial language and associated reference frame has been shown to be affected by the features of the objects in the visual scene, as well as their relative location. When an object does not possess a salient axis, such as a ball, the intrinsic reference frame is usually unavailable for use (see Landau, 1996).^1^ Other constraints on reference frame choice governed by the objects to be described include knowledge regarding functional relations (e.g., Carlson-Radvansky and Radvansky, 1996; Coventry and Garrod, 2004), the perception of other people’s actions with respect to the objects to be described (Tversky and Hard, 2009), and the context in which those objects occur (Mainwaring et al., 2003). For example, imagine a picture of a mail carrier on a page to the left of a picture of a mailbox. Carlson-Radvansky and Radvansky (1996) found that when the mail carrier was shown facing the mailbox, participants preferred to use *in front of* (intrinsic reference frame) compared with *to the left of* (relative reference frame) to describe the mail carrier’s position and vice versa when the mail carrier was facing away from the mailbox. The selection of a spatial reference frame is also influenced by previous discourse (Watson et al., 2004; see also Schober, 1998; Johannsen and De Ruiter, 2013a,b; Dobnik et al., 2014). For example, Watson et al. (2004) found that participants were 10% more likely to use the intrinsic frame if they heard a confederate use that reference frame on the preceding trial, than when the confederate used a relative frame on the preceding trial. Reference frame use is also affected by the non-selection of an available frame for the trial just previously seen by the same participant (Carlson-Radvansky and Jiang, 1998).

**FIGURE 2:**
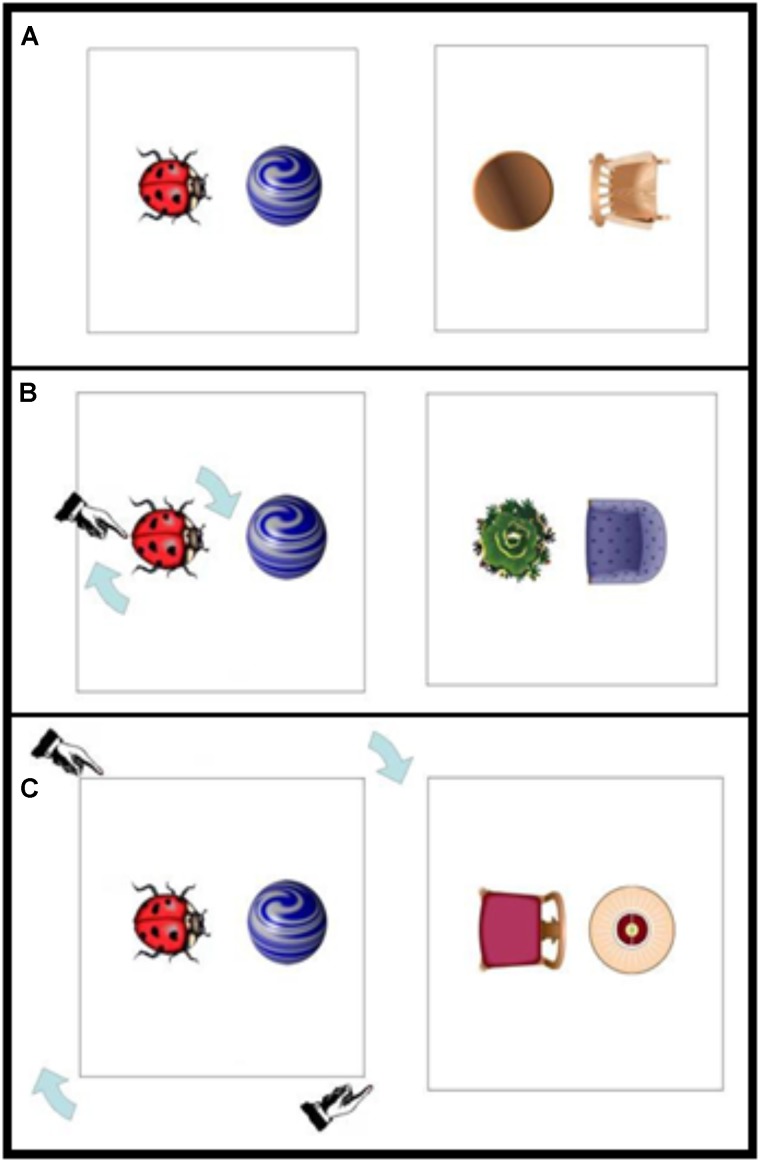
Example materials: **(A)** no visual cue, **(B)** intrinsic visual cue, and **(C)** relative visual cue condition. Right panels show example probe trials. Note that the prime and probe trials consisted of different counterbalanced positions of located object (on the left or on the right relatively) and orientation of reference object (e.g., the ladybug facing toward or away from the located object).

In the Experiments reported below, we first investigate the possible impact of visual motion cueing on the choice of reference frame. This idea not only extends visual constraints on syntactic structure (e.g., Gleitman et al., 2007) to conceptual description, but also affords examination of how changes in the spatial world may draw attention to objects, affecting spatial description. Consider the images shown in **Figure 2**. Imagine that a previous speaker is unsure about which way round the ladybug should be on the card, and spins the ladybug around on its axis until the correct position is determined. This may draw attention to the reference object, and hence to the intrinsic axis of the ladybug (i.e., visually cueing the intrinsic frame). Or instead, imagine that the unsure speaker rotates the whole card (ladybug plus marble), thereby visually cueing the relative frame. Experiment 1 tests exactly this contrast, that is, whether visual cueing – in the form of changing object positions – affects reference frame choice in spatial description.

Previous research on visual cueing has focussed on the influence of static cues (variants of the paradigm originally developed by Posner, 1981) on syntactic (structural) choice in language production (for a review see Myachykov et al., 2011). Visual cues in this context are taken to facilitate the selection between response alternatives, such as selecting between passive versus active sentence structures to describe a picture. In a similar vein, one can argue that different *conceptual* reference frames as expressed through language are available for use (see Carlson-Radvansky and Logan, 1997), and that visual motion cueing may affect the selection of available frames in an analogous way to visual cueing in a structural context.

The first Experiment also examines the interplay between visual cueing and past linguistic description. There is some debate in the literature regarding the influence of visual cues in the presence of a linguistic (structural) cue. While Bock et al. (2004) and Kuchinsky and Bock (2010) have argued that visual cueing only affects choice of language structure in the absence of prior linguistic description, Myachykov et al. (2012) have recently shown that visual cues affect choice of passive versus active sentence productions even when a linguistic cue to structural choice is available. Here we ask if reference frame selection may similarly be influenced by multiple constraints, and if visual cueing persists even when linguistic cues are present.

Building on the results of Experiment 1, Experiment 2 uses eye tracking to test whether language associated with distinct reference frames draws attention to the visual world in differentiable ways. Just as visual cueing may result in changes in spatial description due to their association with different conceptual reference frames, in Experiment 2 we ask if spatial descriptions and their associated reference frames may trigger (or reflect) different attentional patterns when looking at a visual scene on hearing that description. Moreover, we test if these attentional patterns, manifest through eye movement patterns, may persist when looking at new scenes prior to describing them. To preview the results, we find that visual cueing affects choice of reference frame as expressed through language even when prior linguistic description is available (Experiment 1), and that language expressing reference frames is also associated with differential attention paid to the objects in a visual scene as a function of reference frame type (Experiment 2).

## Experiment 1

In this experiment, we examined changes in object position as a possible form of visual cueing on participants’ choice of spatial reference frame, relative to the effects of linguistic cues. To do so, we adopted a between-participants design in which each participant heard one example trial prior to producing spatial descriptions across three (probe) trials. This method was chosen in preference to a within-participant design, which is typically used in confederate priming paradigms (e.g., Branigan et al., 2000; Cleland and Pickering, 2003; Watson et al., 2004) so that participants would not suspect intentional manipulation of the visual cues (and also to avoid the problems with excessive use of confederates in within-participant designs; see Kuhlen and Brennan, 2013 for discussion).

In our study, the experimenter explained that participants would see three pictures, and that they were to describe, as naturally as possible, where one object (the located object) was with respect to a second object (the reference object). The reference object was attached to each trial card using a pin, allowing a rotation manipulation in which the entire scene or the reference object individually, could be rotated. There were three visual cueing conditions. Prior to giving a spatial description, the experimenter either rotated the whole scene (the relative visual cue condition), or rotated just the reference object (the intrinsic visual cue condition), or performed no rotation (the control condition). Rotations occurred at the beginning of each trial and were the same for all participants within the same condition. We hypothesized that rotation of the reference object alone would draw attention to the reference object and its axes, leading to an increased likelihood of an intrinsic description being used to describe the spatial relation between objects (compared to a *no rotation* control). In contrast, we expected rotation of the whole scene to lead to an increased likelihood of a relative frame as we expected a more even distribution of attention across the two objects. Also, since changing the orientation of the whole scene alters their spatial relationship relative to the viewer, this makes the relationship relative to the viewer more salient than the intrinsic relationship between the objects (which is unaffected by this rotation).

In order to have a sense of the extent of visual cueing using this novel paradigm, we also included different spatial description conditions to establish the effects of visual cueing relative to linguistic cueing by an (explicit) example spatial description from the experimenter. The experimenter showed participants an example scene (see **Figures 2A–C**), where an indication of the type of description was given: *“For example, one could say that the marble is blah blah blah the ladybug”* (neutral condition). In this condition, it was clear that a spatial expression was missing, therefore providing a better control than a non-language control condition. In the two explicit example conditions, the experimenter said either *“For example, one could say that the marble is in front of the ladybug”* (intrinsic example) or *“For example, one could say that the marble is to the right of the ladybug”* (relative example). Following the example trial, participants were presented with three probe trials (see **Figure 2**) and were asked to describe where the located object was with reference to the other object for each of the those trials. The reference object was always oriented facing left or right (with the front/back), so for each probe relative and intrinsic descriptions could be used appropriately. Thus, the experiment employed a 3 (visual cueing: no cue, intrinsic cue, relative cue) × 3 (verbal example: neutral, explicit intrinsic example, explicit relative example) between participants design.

### Method

#### Participants

Participants (*N* = 406; 194 male, 212 female, age range 16–70) were a mixture of students recruited from Bremen University and (adult) visitors to the Science Centre in Bremen (Germany) (see **Table 1** for demographics by condition). All participants were native (L1) German speakers and took part on a voluntary basis [no information regarding other (L2) languages spoken was collected].

**Table 1:** Breakdown of participants across locations and conditions in Experiment 1.

Experiment location	Rotation condition	Demographics	Intrinsic reference frame	Relative reference frame	Neutral reference frame
Museum	Scene rotated	*N*	46	45	21
		Gender	20 female	19 female^[2]∗^	15 female
		Age: *M (SD)*	40.87 (12.25)	40.28 (12.18^[2]^)	35.71 (13.55)
	Object rotated	*N*	32	33	22
		Gender	20 female	21 female	6 female
		Age: *M (SD)*	40.97 (11.41)	40.69 (11.92)	29.05 (12.48)
University	No rotation	*N*	58	60	56
		Gender	34 female^[1]^	30 female^[2]^	29 female
		Age: *M (SD)*	28.41 (7.4)	27.88 (8.6)^[2]^	28.11 (8.93^[1]^)

*^∗^Values between square brackets in superscript represent the number of participants in that group for which the relevant demographic information is missing.*

#### Stimuli and Procedure

Participants were approached by the experimenter (a male research assistant), and were asked if they would take part in a 5-min study examining how people describe simple pictures. The experimenter then sat down in a chair next to and pointing in the same direction as the participant (so that the relative frames of experimenter and participant were aligned). The task was then explained. All scenes were presented on cards 21 cm × 21 cm in size, which were held by the experimenter and placed in front of the participant on the table. The example picture showed a ladybug and a marble in one of four relative orientations (counterbalanced across participants) in which the marble was placed either on the right or on the left of the ladybug (looking at the picture), and the ladybug was either facing toward or away from the marble. The probe pictures were composed of common items found in a living room, and there were six different object combinations in the probe set (plant/folding chair, flower bouquet/cocktail chair, carpet/dining chair, Christmas tree/lounge chair, waste-paper basket/living room chair, table/rocking chair). All objects were shown in plan view (see **Figure 2**). The experiment was conducted in German, and the nouns used were all unique lexical items (Pflanze/Klappstuhl, Blumenstrauß/Cocktailsessel, Teppich/Esszimmerstuhl, Weihnachtsbaum/Clubsessel, Papierkorb/Wohnzimmersessel, Tisch/Schaukelstuhl).

Each participant saw one example followed by three (probe) trials. For each probe trial, the experimenter asked “wo ist der/die NOUN in Bezug auf den NOUN?” – i.e., “where is the NOUN (name of located object) in relation to the NOUN (name of reference object)?”.^2^ The located object never had an identifiable front or back (e.g., a plant viewed from above). No participant saw or described the same configuration (i.e., relative positions and orientations of objects) or the same pairs of objects more than once, and the order of pictures was counterbalanced across participants and conditions. All responses were audio recorded, with participants’ consent. For the visual cueing conditions, both the example and the probe trials were preceded by the experimenter rotating either the reference object or the whole picture (360° clockwise and then anti-clockwise), arriving at the desired orientation of objects for that trial. The experimenter did this without making any comment, but from the participants’ perspective it appeared as if the experimenter was unsure as to which way round the card/reference object should be positioned. On debrief, it was confirmed that the unsure experimenter paradigm was successful; no participants reported that they thought the cards/objects were being rotated intentionally, and none of them thought it affected their choice of spatial description. Rotations were conducted at the start of both example and probe trials as we expected participants to be self-primed verbally from probe to probe (see Vorwerg, 2009), and therefore, we also aimed to keep the visual information constant for all probes.

#### Coding of Responses and Data Analyses

We categorized participants’ responses for each probe trial as using either the intrinsic frame, the relative frame, or “other” which did not specify a reference frame (e.g., use of “near”). More complex descriptions than NOUN PREPOSITION NOUN were coded on a first-mention basis consistent with previous studies in both spatial and non-spatial domains (MacWhinney, 1977; Richards et al., 2004). We excluded data from participants who provided a verbal description for the example scene prior to the experimenter producing the example or prior to the rotation by the experimenter for the example scene (*N* = 21), or who produced an invalid response on the first probe trial (*N* = 12), which left 373 participants with usable data.

The use of “other” terms was minimal (5.9% of trials overall); hence, we excluded “other” terms in our analyses. We analyzed the frequency of intrinsic and relative frame terms for the first probe, the second probe, and the third probe separately. The analyses for each of the probe trials were conducted in two ways. A first set of analyses was conducted on all valid responses on the experimental probes, and a second set of analyses was conducted after eliminating data points in which participants could have been lexically primed by the experimenter’s example rather than influenced by the reference frame use alone (Cleland and Pickering, 2003). For example, in **Figure 2**, the use of *the marble is to the right of the ladybug* by the experimenter followed by the scene on the right in **Figure 2C** as first probe trial could prime participants to use the same lexical term (*right of*), thus leading to a conflation of lexical choice with reference frame choice. Such cases, where the example term overlapped with a potentially valid description of the scene (29.6% of all valid probe 1 responses) were dropped from the second set of analyses. This allowed us to examine the effects of prior verbal description on participants’ choice of reference frame independently of (potential) lexical priming. (We were aware of this issue prior to testing, but due to the setup of the experiment, these cases were inevitable.)

### Results

**Table 2** shows the frequency of reference frame use for each probe by condition.^3^ As can be seen in the Table, when there is no visual motion cue or linguistic example, there is a preference for participants to choose the intrinsic reference frame in their descriptions, with a mean of 53–65% intrinsic frame use across probes (with a significant preference for the intrinsic frame for probe 3 only, *χ*^2^(1) = 4.74, *p* = 0.030).

**Table 2:** Number of participants using spatial descriptions involving intrinsic (Int) and relative (Rel) frames (with percentages in brackets) for each probe for each condition in Experiment 1. (Note that the use of other types of description were excluded.)

	Probe 1		Probe 2		Probe 3	
*Prior Description*	Int	Rel	Int	Rel	Int	Rel
**No Visual cue**						
Intrinsic	44 (83%)	9 (17%)	45 (87%)	7 (13%)	47 (87%)	7 (13%)
Relative	15 (25%)	45 (75%)	17 (29%)	41 (71%)	18 (32%)	38 (68%)
Neutral	27 (53%)	24 (47%)	34 (63%)	20 (37%)	35 (65%)	19 (35%)
	86 (52%)	78 (48%)	96 (59%)	68 (41%)	100 (61%)	64 (39%)
**Intrinsic Visual cue**						
Intrinsic	26 (90%)	3 (10%)	28 (88%)	4 (12%)	28 (88%)	4 (12%)
Relative	13 (42%)	18 (58%)	19 (59%)	13 (41%)	21 (70%)	9 (30%)
Neutral	19 (86%)	3 (14%)	19 (90%)	2 (10%)	18 (90%)	2 (10%)
	58 (71%)	24 (29%)	66 (78%)	19 (22%)	67 (82%)	15 (18%)
**Relative Visual cue**						
Intrinsic	40 (89%)	5 (11%)	38 (93%)	3 (7%)	39 (95%)	2 (5%)
Relative	9 (20%)	35 (80%)	3 (8%)	33 (92%)	12 (28%)	31 (72%)
Neutral	5 (31%)	11 (69%)	9 (50%)	9 (50%)	8 (44%)	10 (56%)
	54 (51%)	51 (49%)	50 (53%)	45 (47%)	59 (58%)	43 (42%)

We next analyzed the data for each of the probe trials using generalized linear (logit) models with binomial distributions. Analyzing each probe trial rather than collapsing across the three trials each participant saw was appropriate given that our dependent variable was binomial (Jaeger, 2008) and it allowed us to test whether the effects persisted across trials. Participants either used intrinsic or relative descriptions, as we excluded the small percentage of cases in which “other” terms were used.

For each of the probes we first considered the model fit for the fixed effects (main effects and interaction) in the mixed logit model. For each probe the model with two main effects and interaction (*N* = 373) produced the best fits (log likelihoods of 109.85 for probe 1, 102.89 for probe 2, 97.27 for probe three, all *p* < 0.0001).

The analysis of the first probe produced a main effect of visual motion cueing, *χ*^2^(2) = 10.31, *p* = 0.006. Overall, 71% of participants used an intrinsic description in the intrinsic visual cue condition, as compared with 52% in the no visual cue condition, *χ*^2^(1) = 8.20, *p* = 0.004, and 51% in the relative visual cueing condition, *χ*^2^(1) = 7.14, *p* = 0.008. Not surprisingly, there was a significant main effect of explicit verbal example, *χ*^2^(2) = 69.11, *p* < 0.00001, with 86% of participants using the intrinsic frame in the explicit intrinsic example condition, 57% in the neutral condition, and 27% in the explicit relative example condition (all contrasts, *p* < 0.00001). The interaction was not significant, *χ*^2^(4) = 6.63, *p* = 0.228. The intercept was significant, *χ*^2^(1) = 9.635, *p* = 0.002.

The analysis of the second probe again produced a main effect of visual cueing, *χ*^2^(2) = 8.36, *p* = 0.015, with the same pattern as probe 1; 78% of participants used an intrinsic description in the intrinsic visual cue condition, as compared with 59% in the no visual cue condition *χ*^2^(1) = 8.99, *p* = 0.003 and 55% in the relative visual cue condition *χ*^2^(1) = 10.10, *p* = 0.001. There was also a significant main effect of explicit verbal example, *χ*^2^(2) = 69.00, *p* < 0.00001, but again the interaction between visual cueing and explicit verbal example was not reliable, *χ2*(4) = 7.29, *p* = 0.121. The intercept was significant, *χ*^2^(1) = 26.59, *p* < 0.0001.

For the third probe, there were main effects of visual cueing, *χ*^2^(2) = 8.27, *p* = 0.006 and explicit verbal example, *χ*^2^(2) = 46.12, *p* < 0.00001, with the same pattern observed for probes 1 and 2. This time, however, the interaction between visual cueing and explicit verbal example was also significant, *χ*^2^(4) = 9.99, *p* = 0.04. Examining visual cueing under each of the explicit verbal example conditions revealed effects of visual cueing in the presence of an explicit relative example, *χ*^2^(2) = 15.40, *p* < 0.0001, and in the absence of an explicit example, *χ*^2^(2) = 12.40, *p* = 0.002, but not when the experimenter used an explicit intrinsic example description, *χ*^2^(2) = 1.65, *p* = 0.439. The intercept was significant, *χ*^2^(1) = 33.55, *p* < 0.0001.

A second set of analyses was conducted for each probe trial, this time excluding any trials where participants could have been lexically primed by the experimenter’s initial description. These analyses produced the same pattern of results as above (with the same pattern of significance).

The results thus far indicate that visual motion cues exert a strong influence on reference frame choice even in the presence of linguistic example descriptions, with reliable differences between the intrinsic visual cue condition and both the relative and neutral visual cue condition across all probes (all *p* < 0.01). To more finely determine the influence of visual motion cueing, we further examined visual cueing with participants who were not primed with a specific reference frame (as expressed through language). For each probe this produced significant effects of visual motion cueing condition [probe 1, *χ*^2^(2) = 10.46, *p* = 0.005; probe 2, *χ*^2^(2) = 6.60, *p* = 0.037; probe 3, *χ*^2^(2) = 7.58, *p* = 0.023]. For each probe there was a significant difference between the intrinsic visual cueing condition and the other two conditions (all *p* < 0.01), but the difference between the relative visual cueing condition and the neutral condition was not significant for any of the probe trials (all *p* < 0.05).

### Discussion

This experiment has produced the first evidence that reference frame choice as expressed in language can be visually cued. In the no visual cue/no linguistic example condition 53% of people used the intrinsic frame for the first probe, but with an intrinsic visual cue this figure jumped to 86% and fell to 31% with a relative visual cue in the absence of a verbal example. On their own, these effects are similar in magnitude to the effect of an explicit linguistic example in the absence of a visual cue (83% intrinsic frame use following an explicit intrinsic example on the first probe; 25% intrinsic frame use following an explicit relative example). This shows that visual cueing does exert a powerful influence on spatial description choice.

The unintentional visual motion manipulation provides strong evidence that reference frame choice as expressed in language can be driven by an unconscious cueing mechanism. Participants were unaware that the experimenter intended to perform the rotations in the intrinsic and relative visual cue conditions, and did not report attending to the rotations, or that they affected their language choice in any way. Moreover, the results extend effects of visual cueing on language production from syntactic structure to conceptual description.

The results of visual cueing were significant comparing the intrinsic motion cueing condition relative to the other conditions, but there is no evidence for a reduction in intrinsic frame use, and a corresponding increase in relative frame use, with a relative visual motion cue compared to the neutral condition. One explanation for this has to do with a general preference for intrinsic frame use in this experiment in the neutral condition; a visual cue can increase the likelihood of using the preferred frame, but switching away from the preferred frame may be much harder. Alternatively, it may be argued that rotating the reference object in the intrinsic visual cueing condition may unambiguously draw attention to the reference object, with the rotation of the whole card being a more ambiguous attentional manipulation as it does not direct attention to a specific object, but rather to changes in relative locations.

The inclusion of explicit example descriptions from the experimenter in this experiment has also provided evidence that visual cueing can affect spatial description in the face of such example descriptions. This was the case both for probes 1 and 2 – where there was a main effect of visual cueing and no interaction between visual cueing and explicit example condition – and for probe 3 where the interaction showed an effect of visual cueing in the presence of an explicit relative example or where an explicit example was not given. The absence of an effect of visual cueing in the presence of an explicit intrinsic example on probe 3 is likely to be a reflection of the general preference for intrinsic descriptions as we noted above – with 60% intrinsic descriptions produced across probes in the no visual cue/no explicit example condition, an explicit intrinsic example pushed this general preference to ceiling. More broadly, however, the persistence of visual motion cueing in the face of a past linguistic example is consistent with the results of Myachykov et al. (2012), who measured the impact of visual cueing and linguistic cueing on transitive sentence production (choice of passive or active).

Next, we extend the effects of visual cueing to consider whether reference frames, as expressed in spatial description, lead to different ways of visually attending to the world. Just as manipulating objects in the world leads to changes in preferences for reference frames as expressed in spatial language to describe object location, we hypothesized that language in turn may lead to different attentional patterns when looking at visual scenes.

## Experiment 2

There is substantial evidence from eye tracking studies that language drives attention to parts of the spatial world. In particular, attention is directed to objects as language unfolds in real time (e.g., Eberhard et al., 1995; Tanenhaus et al., 1995; Allopena et al., 1998), with evidence of anticipatory eye movements (e.g., Knoeferle and Crocker, 2006; Altmann and Kamide, 2007). In relation to spatial language, Chambers et al. (2002) found that upon hearing, for example “*put the cube inside the can*,” participants used information from the preposition (*inside*) to restrict the referential domain to objects with relevant spatial properties (e.g., container-like objects with the right dimensions to contain the to-be-moved object) prior to hearing the referent at the end of the sentence.

Despite the extensive literature on spatial reference frames as expressed in language, there is no current evidence to suggest that these reference frames are associated with processing visual scenes in different ways. Models of spatial language processing (e.g., Regier and Carlson, 2001) assume that attention is directed from a reference object to a located object, and the issue of different attentional patterns associated with different reference frames has not been considered. Following the visual cueing results from Experiment 1, we hypothesized that reference frames expressed through language might also affect how attention is allocated to a located object versus a reference object within a scene, and potentially how attention is allocated within a reference object. We had two hypotheses regarding what happens upon hearing prepositional phrases associated with different reference frames. While intrinsic descriptions require assigning direction from the reference object by first identifying the relevant parts and intrinsic axes of the object, the features of the reference object are not relevant with relative descriptions. Therefore, we predicted, firstly, that more time would be spent looking at the reference object compared to the located object following intrinsic descriptions, compared to relative descriptions. Secondly, we speculated that more time would be spent looking at the front and back of the reference object compared to the middle of the object following intrinsic descriptions compared to relative descriptions.

If distinct eye movement signatures are present following the example description, we also wanted to test whether such eye movement patterns persist when looking at a new scene prior to producing a new description. This would provide evidence that language drives eye movement patterns (i.e., visual attention), which in turn might drive future spatial description choice. Such a mechanism offers a low effort and automatic means with which alignment with an interlocutor could emerge without the requirement for high-level conscious intention, which would be effortful (cf. Kahneman, 2012). This approach is not without precedent: It has long been established that participants fixate objects approximately 900 ms prior to mentioning them (Griffin and Bock, 2000), and that cueing objects – whether explicitly or implicitly – affects the likelihood of object mention (and hence choice of syntactic structure; e.g., Gleitman et al., 2007). Subsequently, these findings were expanded to show that the scan paths participants exhibit when looking at a picture prior to description are predictive of the types of syntactic structures participants use in describing pictures (Coco and Keller, 2012).

### Method

#### Participants

Participants were 84 students from the University of East Anglia (28 male, 56 female, age range 18–50 [*M* = 21.89, *SD* = 5.15]), who took part for course credits or monetary payment. (**Table 3** shows the demographics by condition.) All participants were monolingual (or near-monolingual), native (L1) English speakers.

**Table 3:** Demographics by condition.

	Intrinsic	Relative	Neutral
*N*	21	19	42
Gender	14 female, 7 male	11 female, 8 male	29 female, 13 male
Age: range (*M, SD*)	18–28 (20.43, 2.1)	18–50 (22.84, 7.54)	18–41 (22.29, 4.88)

#### Materials and Setup

**FIGURE 3:**
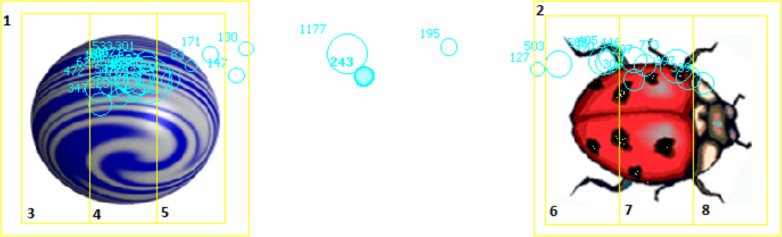
Interest areas in Experiment 2. Left areas: 3, 6; Middle areas: 4, 7; Right areas: 5, 8.

Participants were placed in front of the eye tracker. On the screen, simple spatial scenes consisting of two objects were shown in the same combinations as in Experiment 1 (see **Figure 2**). For every object, four interest areas (IA) were assigned (**Figure 3**). An IA was assigned to the whole object, and each object was further divided into three equal parts (left, middle, right). This allowed us to monitor both the objects and the areas within the objects participants were looking at prior to producing their spatial descriptions.

Following Experiment 1, three different, randomly assigned versions were used in an example trial: a relative reference frame (“The marble is to the left/right of the ladybird”), an intrinsic reference frame (“The marble is in front of/behind the ladybird”) or no reference frame (neutral condition: “The marble is ‘blah blah’ the ladybird”). Note that all priming was verbal, there was no visual cueing in Experiment 2.

After the example trial, six different spatial scenes were presented as probe trials. (An additional three probe trials were included, compared to Experiment 1, in order to examine how long lasting the effects of an example description might be.) Six different probe trial scenes using two objects (one reference, one located object, e.g., a lounger and a Christmas tree, a sofa and a lamp, etc.) were used, and each probe trial was prepared in 4 conditions: located object to the left/right of the reference object, and the reference object facing toward/ away from the located object. The probe trials were pseudo-randomized so that the located object was located 3 times on each side (to the left/right) of the reference object, and so that each orientation of the reference object (facing toward/away from the located object) occurred at least once in each left/right position. The order of the probe trials (and the exact orientation per probe trial) was pseudo-randomized over 12 different lists, to eliminate the possibility of order-effects. Every participant saw each different scene (in one of the orientations) at a random point throughout the probe trials. The reference object in the first probe trial had the opposite location compared to the prime trial, but the same orientation as the prime trial. For example, if the ladybird was presented to the left of the marble in the prime trial, the reference object of the first probe trial would be to the right; whereas if the reference object was oriented to the right, this was the same in the probe trial. Therefore, the term used in the instruction was invalid for the first probe trial, preventing the lexical priming we discussed in relation to Experiment 1. The participants were instructed to verbally describe the situation, and their response was automatically recorded and saved as a wav file.

Eye movements were recorded with an SR research Eyelink 1000 eye tracker sampling at 1000 Hz (SR Research Ltd., Ottawa, ON, Canada). Viewing was binocular, but only the position of one eye was tracked per participant. Stimulus presentation was programmed using SR research Experiment Builder software. The eye tracker and a 19″ CRT display monitor (refresh rate of 140 Hz) were interfaced with a 3-GHz Pentium 4 PC, which controlled the experiment and logged the position of the eye throughout the experiment. Throughout the task, participant’s heads were stabilized with the use of a chinrest.

#### Procedure

At the beginning of the experiment, eye position was calibrated and validated using a nine-point sequence. Instructions were presented to the participants both verbally (spoken by a native English narrator) and visually on the screen. Then the objects used in the study were presented so that participants would know the labels for each object, and could easily identify the objects when describing them. At the start of the experiment an example trial was presented (same as Experiment 1), followed by six probe trials. The order of trials was pseudo-randomized across 12 different lists. Participants were asked to look at each picture, wait for the question (Where is object A in relation to object B), and then answer with a full sentence.

The design consisted of a single variable with 3 levels (example trial: intrinsic, relative, neutral), and it was manipulated between participants. The dependent variables were the IA participants looked at on the example and the probe trials (i.e., time spent looking at the located object, the reference object, and parts of the reference object), and the type of reference frame produced by participants in the probe trials.

### Results

We first examined whether the linguistic example affected the choice of reference frame as expressed in language across the six probe trials, following the same coding and data analysis strategy used in Experiment 1. **Table 4** shows the frequency of reference frame use for each probe by condition. Note that all participants in this experiment used an intrinsic or relative description (except for one or two trials where the microphone failed to record the response).

**Table 4:** Number of participants using spatial descriptions involving intrinsic (Int) and relative (Rel) frames (with percentages in brackets) for each probe for each condition in Experiment 2.

	Probe 1		Probe 2		Probe 3		Probe 4		Probe 5		Probe 6	
	Int	Rel	Int	Rel	Int	Rel	Int	Rel	Int	Rel	Int	Rel
Intrin.	16 (76)	5 (24)	18 (86)	3 (14)	18 (86)	3 (14)	18 (86)	3 (14)	18 (86)	3 (14)	18 (86)	3 (14)
Relat.	3 (16)	16 (84)	4 (21)	15 (79)	7 (37)	12 (63)	7 (37)	12 (63)	7 (37)	12 (63)	6 (32)	13 (68)
Neu.	8 (19))	34 (81)	8 (19)	34 (81)	10 (24)	32 (76)	13 (31)	29 (69)	13 (31)	29 (69)	16 (38)	26 (62)

*Note that the use of other types of description were excluded. Intrin., Intrinsic linguistic example condition, Relat., Relative linguistic example, and Neu., Neutral.*

The analysis of the six probe trials, using generalized linear (logit) models with binomial distributions (with similar model fits to Experiment 1), produced significant effects of linguistic example for all six probe trials, with *χ*^2^(2) values ranging from 12.36 to 20.79 (all *p’s* < 0.01). For each probe intrinsic descriptions were used more in the intrinsic example condition (ranging from 76 to 86%), compared to the relative example condition (intrinsic description use ranged from 21 to 37%) or the neutral condition (range from 19 to 38% intrinsic description use). For all probe trials, there was a reliable difference between the intrinsic condition and the other two conditions (all *p’s* < 0.0001), but never between the relative condition and the neutral condition (all *p’s* > 0.05).

**FIGURE 4:**
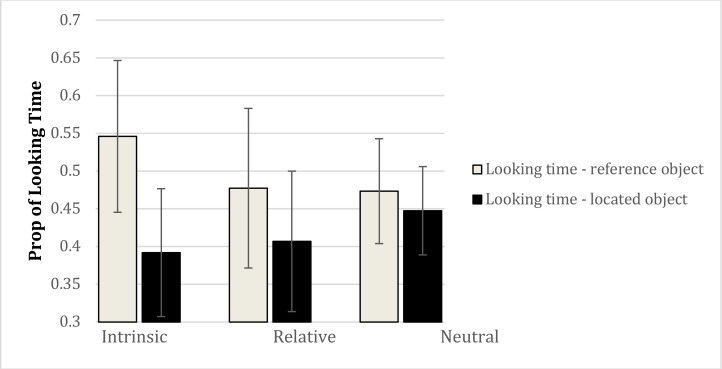
Interaction between condition and object with respect to looking time in Experiment 2. Error bars represent 95% confidence intervals.

We next examined the eye movement data. In order to examine the time spent attending to the reference and located objects across the probe and prime trials, we summed the fixation durations for the reference and located objects separately from the onset of the question (*“Where is the marble in relation to the ladybird”*) in the example and probe trials to the end of the trial for the example trial, and to the beginning of the response (onset of spatial description) for the probe trials. These data were analyzed with a 7 × 3 × 2 (trial: example, probes 1–6) × (condition: intrinsic, relative, neutral) × (object: reference, located) mixed model ANOVA. This produced a main effect of object, *F*(1,81) = 25.34, *p* < 0.00001 ($\eta_{\text{p}}^{2}$ = 0.238). Overall, more time was spent looking at the reference object (*M* = 49.99%) compared to the located object (*M* = 41.54%). The interaction between condition and object was also significant, *F*(2,81) = 5.80, *p* < 0.005 ($\eta_{\text{p}}^{2}$ = 0.125) (see **Figure 4**). More time was spent looking at the reference object in the intrinsic condition (*M* = 54.60%) compared to both the neutral (*M* = 47.34%) and relative (*M* = 47.73%) conditions (both *p’s* < 0.008, LSD tests). The difference between the neutral and relative conditions was not reliable (*p* = 0.86). In contrast, less time was spent looking at the located object in the intrinsic condition (*M* = 39.12%) compared to the neutral condition (*M* = 44.74%). There was also a main effect of trial, *F*(6,486) = 3.01, *p* < 0.01 ($\eta_{\text{p}}^{2}$ = 0.036), revealing less time looking at the located and reference objects during the example trial compared to the probe trials, but critically, trial did not interact with condition or object, and the three-way interaction between trial, condition, and object was also not significant (*F* < 1.0).

This analysis shows that looking behavior across conditions was consistent across example and probe trials, consistent with the view that eye movement patterns are maintained from the linguistic example across all probe trials.

We next analyzed the amount of time spent looking at the parts of the reference object, separating areas that are diagnostic (front and back) vs. not diagnostic (center) of object orientation. In order to do so, we summed the fixation durations at the front (one-third of the object) and back (one-third of the object) of the reference object and subtracted the time spent looking at the middle (one-third of the object). A 3 × 7 (condition: intrinsic, relative, neutral) × (trial: example, probes 1–6) mixed model ANOVA showed no main effect of trial (*F* < 0.6, $\eta_{\text{p}}^{2}$ = 0.007), no main effect of condition *F*(2,81) = 1.62, *p* = 0.20, ($\eta_{\text{p}}^{2}$ = 0.39), nor an interaction between trial and condition (*F* < 0.9, $\eta_{\text{p}}^{2}$ = 0.020). However, relatively more time was spent looking at the front and back of the reference object in the intrinsic condition (*M* = 5.69%) than in either the relative (*M* = 1.28%) or the neutral (*M* = 1.48%) conditions.

### Discussion

In Experiment 2, we again found effects of linguistic example on reference frame choice as expressed in language, and the effect was robust across probe trials. In the neutral condition, there was a strong preference to use the relative frame. When an intrinsic example was used, 84% of probe trials employed intrinsic descriptions, compared to 27% in the neutral condition, and 30% in the relative example condition. As before this represents a considerable influence of previous description on reference frame selection, and this persists across the extended duration of the experiment. What is more informative is the eye movement data regarding how participants looked at visual scenes following the example description and prior to their own descriptions across the probe trials.

With respect to looking times on the reference and located objects, the data provide the first evidence that reference frames, expressed in language, are associated with different visual attentional patterns. Across all the trials (i.e., example trial and six probes) more time was spent looking at the reference object than the located object following the offset of the prepositional phrase (diagnostic of reference frame) when the example trial contained a preposition denoting an intrinsic reference frame compared to a relative or neutral frame. This is consistent with earlier work showing more coarse-grained effects of prepositions on object reference using the Visual World Paradigm (Chambers et al., 2002), and is also consistent more broadly with a range of studies showing the rapid interplay between language and visual attention (for a review, see Henderson and Ferreira, 2004). It is particularly striking that the looking time behavior was consistent across the example trial and six probe trials, just as the responses were consistent across trials.

The analyses of looking behavior to parts of the reference objects did not produce reliable results. This may well be a result of the size of objects used. The mean size of objects on the screen was 5 cm × 5 cm, which means that participants could apprehend the entire object within para-foveal vision, and thus, eye movements to left and right sides of the object were unnecessary to orient front and back. Future studies could increase the size of the objects to investigate how participants establish object axes across multiple fixations.

## General Discussion

In two experiments, we examined effects of visual cueing and prior linguistic descriptions on the types of reference frames people use when describing simple spatial scenes. In Experiment 1, the new visual cueing manipulation exerted a powerful influence on reference frame choice. In Experiment 2, the data are consonant with the view that language draws attention to the visual world in specific ways, consistent with visual cueing. We consider the mechanisms involved below, and implications for theories of language production more generally. However, it is first important to discuss consistencies and differences across the experiments with respect to reference frame use in spatial descriptions.

While sizeable increases in intrinsic frame use were observed across both experiments following an intrinsic description of a spatial scene, it is notable that in the neutral condition there was a preference to use intrinsic descriptions in Experiment 1 (60% of verbal descriptions across probes) and relative descriptions in Experiment 2 (71% across probes). It is worth considering why this was the case. There is much disagreement regarding general preference for reference frames in the literature. For example, some authors have argued that the ease of perceptual availability and reduction in computational effort makes the relative frame dominant/most preferred (e.g., Linde and Labov, 1975; Levelt, 1982, 1989), while others have argued that the intrinsic frame is preferred (Miller and Johnson-Laird, 1976; Carlson-Radvansky and Irwin, 1993; Carlson-Radvansky and Radvansky, 1996; Taylor et al., 1999). Experiment 1 was conducted in German and Experiment 2 in English, which might be a natural place to start when trying to unpack these differences in reference frame preference. However, studies examining differences in reference frame preference across languages have been inconclusive (see Tenbrink, 2007, 2009 for discussion), suggesting that discourse and task context effects can be a much stronger influencing factor than cross-linguistic variation, particularly within closely related cultural and linguistic settings (as in our case). Indeed, reference frame choice has been shown to be affected by a range of situational influences, including the embedding of the objects in more complex and real-world scenes (Johannsen and De Ruiter, 2013a,b), and the communicative context in which the speaker is situated (Galati and Avraamides, 2013). In our case, the task was presented in slightly different ways – on a table top in Experiment 1, as opposed to a computer screen in Experiment 2. While we did not set out to test this effect, we note that the ensuing preferences for different reference systems in our neutral condition (without cues) are consistent with findings (in English) by Walker (2013), who showed that changing the orientation of the same scene affects the likelihood of using a relative versus intrinsic description, with substantial increases in relative frame use with vertical presentation compared to horizontal presentation (and vice versa for the intrinsic frame). However, in order to definitively unpack the origins of the differences in the frequencies of reference frame preferences across experiments, further empirical examination of context (including orientation manipulations) is warranted across languages (also taking into account the frequency of use of terms within particular reference frames in individual languages).

Turning to visual cueing, in Experiment 1 the use of the intrinsic frame occurred more frequently in spatial descriptions when the reference object in the scene rotated on its axis, and least when the whole scene rotated, compared to a non-movement baseline. There are two points to make about these particular findings.

First, the rotations we used map directly onto the diagnostic criteria Levinson (1996, 2003) provided for reference frames across languages. The intrinsic frame holds when the whole scene is rotated while the relative frame holds when the reference object is rotated. Drawing attention to changes in the relative frame or changes in the intrinsic frame may prime the use of the relative and intrinsic frames respectively when the objects return to a static and fixed state following rotation. Alternatively, it may be the case that rotating the reference object simply draws more attention to it than to the located object, while changing the positions of both located and reference objects through whole scene rotation draws more equal attention to both objects. Future work would do well to try to tease apart these possibilities; this could be done using exogenous visual cueing (e.g., Gleitman et al., 2007; Myachykov et al., 2012) on neutral (static) scenes to test if the cueing effects persist in the absence of rotation (Gleitman et al., 2007; Myachykov et al., 2012).

Second, the use of the unsure experimenter paradigm produced effects without the participants’ awareness that the rotations were intentional (confirmed at debrief), and without participants being aware that the rotations affected their spatial descriptions. This provides good evidence that visual cueing is taking place at a level where little conscious and strategic processing is taking place.

More broadly, the results of Experiment 1 can be considered in light of recent work showing that drawing attention to the visual world affects syntactic structure in descriptions when the visual objects are cued either explicitly (Tomlin, 1997) or subliminally (Gleitman et al., 2007). The effects of visual cueing have not been considered so far in the context of linguistic descriptions given by an interlocutor. Yet, it is often the case that spatial descriptions occur in tandem with changes to the spatial world in a dialog setting, for example when discussing meteorological data or commenting on sporting events. Our data show that visual cueing affected choice of reference frame in language in the face of the experimenter using their own relative perspective as an example description. These results are consistent with the only other study we know of to cross structural priming and visual cueing - that of Myachykov et al. (2012) – who measured the impact of both variables on transitive sentence production word (choice of passive or active). They also found no interaction between visual cueing and structural priming, albeit in a different language domain. The persistence of visual cueing in the face of explicit linguistic examples suggests that results are not merely limited to monolog settings, paving the way for experiments that more directly cross verbal and visual information to assess how multiple constraints affect language choice.

Our results also extend effects of visual cueing from syntactic structure to the conceptual domain. The choice of spatial language reflects the choice of an underlying frame of reference, which is usually taken to be at a conceptual level of representation rather than a lexical level. In most situations, multiple reference frames are available for use (Carlson-Radvansky and Logan, 1997), and the speaker needs to select between these reference frames for language production. Visual cueing affects this reference frame selection – how one talks about the world *conceptually* - consistent with the influence of past linguistic information on conceptualization (Garrod and Anderson, 1987; Watson et al., 2004).

Experiment 2 provides the first empirical evidence that more time is spent looking at the reference object following an intrinsic description compared to when a neutral or relative description is given. Reference frames have received much attention in the field of spatial language and spatial cognition, but thus far eye-tracking data regarding reference frames has not been available. Not only are reference frames theoretically differentiable (cf. Levinson, 1996), but it would seem that they are associated with differential allocation of attention to the reference and located objects when one looks at a spatial scene following the use of a reference frame in a spatial description by an interlocutor. Moreover, this pattern of differential attention persists when participants look at a different visual scene with a view to then describing it. Since there was no interaction between trial (example trial, probes 1–6) and object (reference, located), the same pattern held between the example and probe trials. Speculatively, one might argue that this finding suggests a possible mechanism to explain so-called “linguistic alignment” effects (cf. Pickering and Garrod, 2004) consistent with visual cueing in Experiment 1. Language draws attention to the world in specific ways, leading to looking at a new spatial scene in the same way. In turn, looking at the world in a particular way, consistent with the effects of endogenous and exogenous visual cueing on choice of syntactic structure (e.g., Gleitman et al., 2007), leads to the increased likelihood of the same type of description being used as that used on the example (prime) trial. Rather than regarding past linguistic description and visual cueing as completely independent and different parameters, one can argue that there is a close interplay between language and visual attention, such that they support each other to maximize alignment between interlocutors. This offers a natural middle ground between the somewhat polarized accounts of linguistic alignment currently in the literature, with strategic accounts at one end (e.g., Clark, 1996) and a supposed low level “priming” mechanism (cf. Pickering and Garrod, 2004) at the other.

Finally, we can return to the range of influences on choice of language considered at the outset of this paper. Consistent with work on the influence of visual (cued) attention on syntactic structure (Gleitman et al., 2007), we have suggested that drawing attention to the intrinsic frame either through an intrinsic prior (example) description or through rotation of the reference object may direct visual attention to the reference object, increasing the likelihood of producing an intrinsic description compared to no-prime or relative-prime conditions. This provides a parsimonious approach to the effect of multiple influences on spatial description, starting with the assumption that language and visual changes can direct the attention of the speaker in similar ways. It also affords a means to test whether a possible strategic route to spatial description, where for example people deliberately choose to ignore past information (systematic misalignment in dialog; Healey, 2008), results in overriding visual attentional patterns (akin to dual process models of semantic priming; see Mummery et al., 1999), or inhibition of the influence of past spatial description in the first instance.

In summary, we have presented the first evidence for visual cueing of reference frames as expressed in language, and for distinguishable looking behavior patterns as a function of reference frames expressed in language. Using between-participants designs where past information can be systematically manipulated immediately prior to a probe trial description provides a clean way of testing how language choice is affected by multiple constraints.

## Ethics Statement

This study was carried out in accordance with the recommendations of the APA, APS, BPS, and in accordance with the Declaration of Helsinki, with informed consent received from all subjects prior to participation. The protocol was approved by the Northumbria University School of Psychology Ethics Committee, the University of East Anglia School of Psychology Ethics Committee, and by the University of Bremen.

## Author Contributions

KC, EA, and TT originated the program of work and designed Experiment 1. KC, HG, and PE designed Experiment 2. KC performed analyses for Experiment 1. KC, HG, and PE analyzed the data for Experiment 2. KC took primary responsibility for drafting the manuscript, with input and review from the other authors. All authors approved submission of the final version of the manuscript prior to submission.

## Conflict of Interest Statement

The authors declare that the research was conducted in the absence of any commercial or financial relationships that could be construed as a potential conflict of interest.

## Footnotes

1 One exception is when the ball is in motion, where the “accidental intrinsic” frame may be used (Jackendoff, 1996). In this case, the front of the ball is the part of the ball, which is always ahead of the rest of the ball in motion.

2 The repertory and usage patterns of German spatial expressions are equivalent to English with respect to the effects targeted here (see Tenbrink, 2009).

3 Across probes individuals were generally consistent in their choice of spatial description, with at least 88% of participants using the same reference frame as expressed in language on the first and last probes in Experiments 1 and 2.
